# Supplementary material for: Bioactive Alkaloids from the Mangrove-Derived Fungus Nigrospora oryzae SYSU-MS0024
Source: Mar Drugs. 2024 May 9;22(5):214. doi: 10.3390/md22050214 (PMC11123012; doi:10.3390/md22050214)
Supplement: Supplementary file 1 [file marinedrugs-22-00214-s001.zip › marinedrugs-2954655-supplementary.docx]

**Supporting Information**

**Bioactive Alkaloids from the Mangrove-derived Fungus *Nigrospora oryzae* SYSU-MS0024**

Xiaokun Chen ^1, †,^ Senhua Chen ^2, †^, Heng Guo ^2^, Xin Lu ^2^, Hongjie Shen ^3^, Lan Liu ^2, 3^, Li Wang ^1^, Bin Chen ^3^, Yi Zhang ^1, 4^, and Yayue Liu ^1, 4*^

1. Guangdong Provincial Key Laboratory of Aquatic Product Processing and Safety, Guangdong Province Engineering Laboratory for Marine Biological Products, Guangdong Provincial Engineering Technology Research Center of Seafood, Key Laboratory of Advanced Processing of Aquatic Product of Guangdong Higher Education Institution, Zhanjiang Municipal Key Laboratory of Marine Drugs and Nutrition for Brain Health, Research Institute for Marine Drugs and Nutrition, College of Food Science and Technology, Guangdong Ocean University, Zhanjiang, 524088, P.R.China; 13129717040@163.com (X.C.) ; wangli991003@163.com (L.W.); hubeizhangyi@163.com (Y.Z.); yayue_liu@163.com (Y.L.)
2. School of Marine Sciences, Sun Yat-sen University, Zhuhai 519000, China; chensenh@mail.sysu.edu.cn (S.C.); hengeguo163@163.com (H.G.); luxin36@mail2.sysu.edu.cn (X.L.); cesllan@mail.sysu.edu.cn (L.L.).
3. Southern Laboratory of Ocean Science and Engineering (Guangdong, Zhuhai), Zhuhai 519000, China; shenhj5@mail2.sysu.edu.cn (H.S.); chenbin@sml-zhuhai.cn (B. C.)
4. Collaborative Innovation Center of Seafood Deep Processing, Dalian Polytechnic University, Dalian 116034, P.R.China

***** Correspondence: yayue_liu@163.com

† These authors contributed equally to this work.

**Table of Contents**

**Figure S1.** The HR-ESIMS spectrum of compound **1**.

**Figure S2.** The ^1^H NMR (400 MHz) spectrum of compound **1** in CDCl_3_.

**Figure S3.** The ^13^C NMR (100 MHz) spectrum of compound **1** in CDCl_3_.

**Figure S4.** The HSQC spectrum of compound **1** in CDCl_3_.

**Figure S5.** The ^1^H-^1^H COSY spectrum of compound **1** in CDCl_3_.

**Figure S6.** The HMBC spectrum of compound **1** in CDCl_3_.

**Figure S7.** The UV spectrum of compound **1**.

**Figure S8.** The IR spectrum of compound **1**.

**Figure S9.** The HR-ESIMS spectrum of compound **2.**

**Figure S10.** The ^1^H NMR (400 MHz) spectrum of compound **2** in CDCl_3_.

**Figure S11.** The ^13^C NMR (100 MHz) spectrum of compound **2** in CDCl_3_.

**Figure S12.** The HSQC spectrum of compound **2** in CDCl_3_.

**Figure S13.** The ^1^H-^1^H COSY spectrum of compound **2** in CDCl_3_.

**Figure S14.** The HMBC spectrum of compound **2** in CDCl_3_.

**Figure S15.** The UV spectrum of compound **2**.

**Figure S16.** The IR spectrum of compound **2**.

**Figure S17.** ^1^H NMR (400 MHz) spectrum of (*S*)-MTPA esters **2** in pyridine-*d*_5_.

**Figure S18.** The ^1^H-^1^H COSY spectrum of (*S*)-MTPA esters **2** in pyridine-*d*_5_.

**Figure S19.** ^1^H NMR (400 MHz) spectrum of (*R*)-MTPA esters **2** in pyridine-*d*_5_.

**Figure S20.** The ^1^H-^1^H COSY spectrum of (*R*)-MTPA esters **2** in pyridine-*d*_5_.

**Figure S21.** The HR-ESIMS spectrum of compound **3**.

**Figure S22.** The ^1^H NMR (400 MHz) spectrum of compound **3** in CDCl_3_.

**Figure S23.** The ^13^C NMR (100 MHz) spectrum of compound **3** in CDCl_3_.

**Figure S24.** The HSQC spectrum of compound **3** in CDCl_3_.

**Figure S25.** The ^1^H-^1^H COSY spectrum of compound **3** in CDCl_3_.

**Figure S26.** The HMBC spectrum of compound **3** in CDCl_3_.

**Figure S27.** The UV spectrum of compound **3**.

**Figure S28.** The IR spectrum of compound **3**.

**Figure S29.** Chiral resolution liquid chromatogram of compound **3**.

**Table S1.** The energy and Boltzmann distribution of the optimized conformers of (5*R*, 1’*R*)-**1,** (5*S*, 1’*R*)-**2,**  and (1’*R*)-**3**.

**Table S2.** Cartesian coordinates of the low-energy reoptimized conformers of (5*R*, 1’*R*)-**1**.

**Table S3.** Cartesian coordinates of the low-energy reoptimized conformers of (5*S*, 1’*R*)-**2**.

**Table S4.** Cartesian coordinates of the low-energy reoptimized conformers of (1’*R*)-**3**.


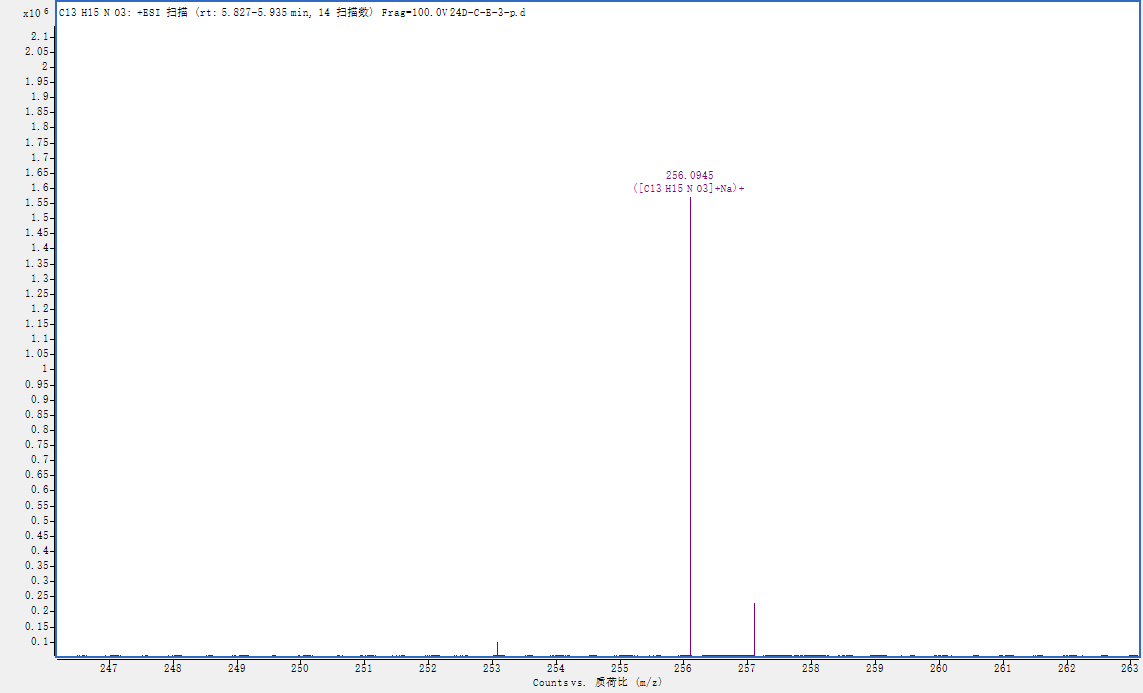


| SPECTRUM - simulation : |  |  |  |  |
| --- | --- | --- | --- | --- |
|  |  |  |  |  |
| *m/z* | Theo. Mass | Delta (ppm) | RDB equiv. | Composition |
| 256.0945 | 256.0950 | 0.48 | 7 | C_13_H_15_NO_3_Na |

**Figure S1.** The HR-ESIMS spectrum of compound **1**.


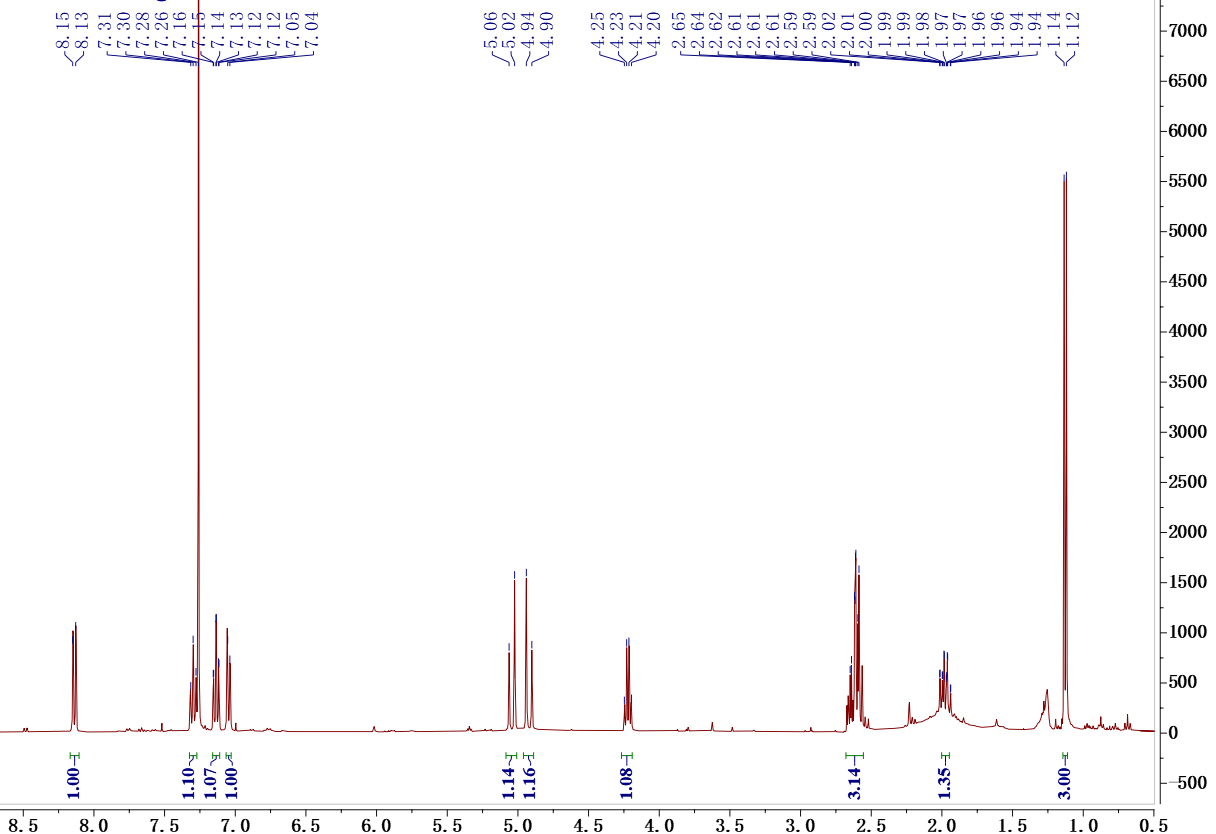


**Figure S2.** The ^1^H NMR (400 MHz) spectrum of compound **1** in CDCl_3_.


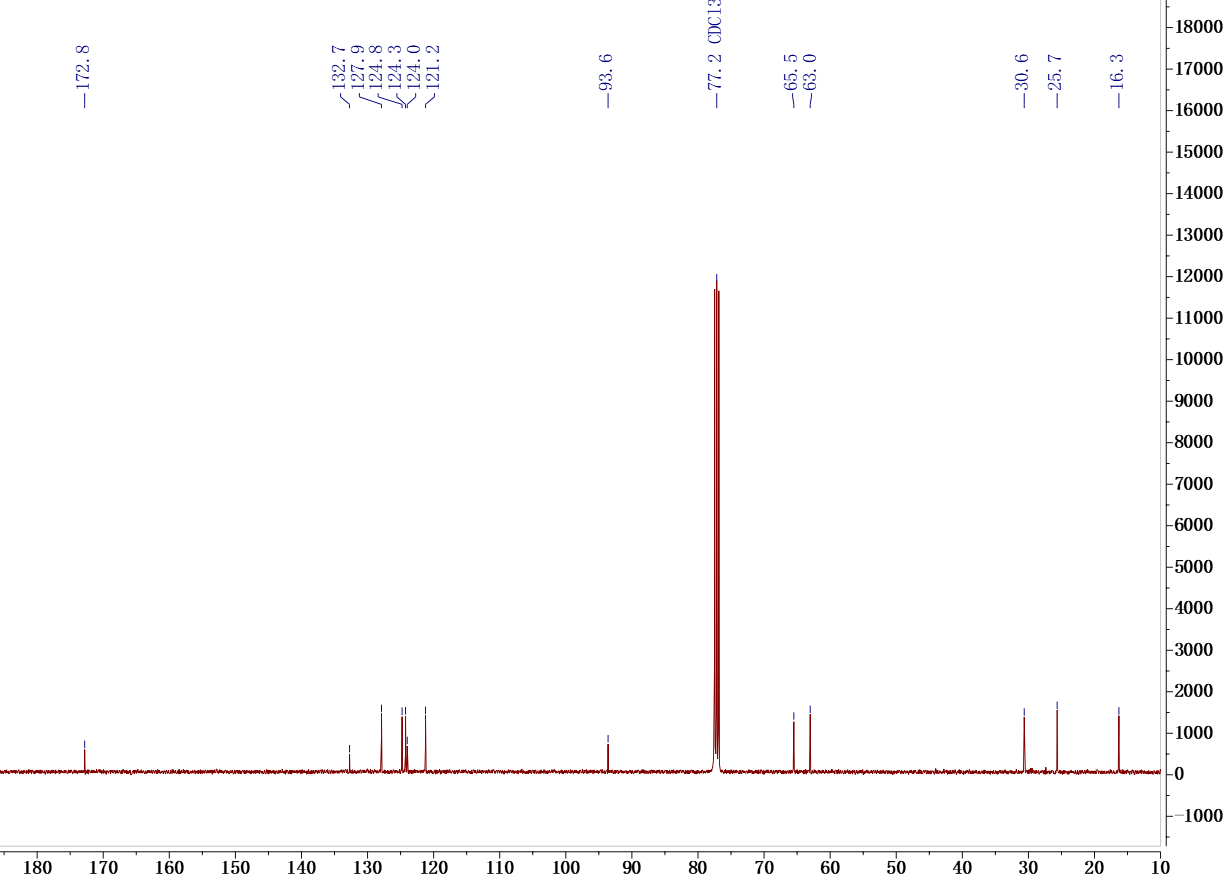


**Figure S3.** The ^13^C NMR (100 MHz) spectrum of compound **1** in CDCl_3_.


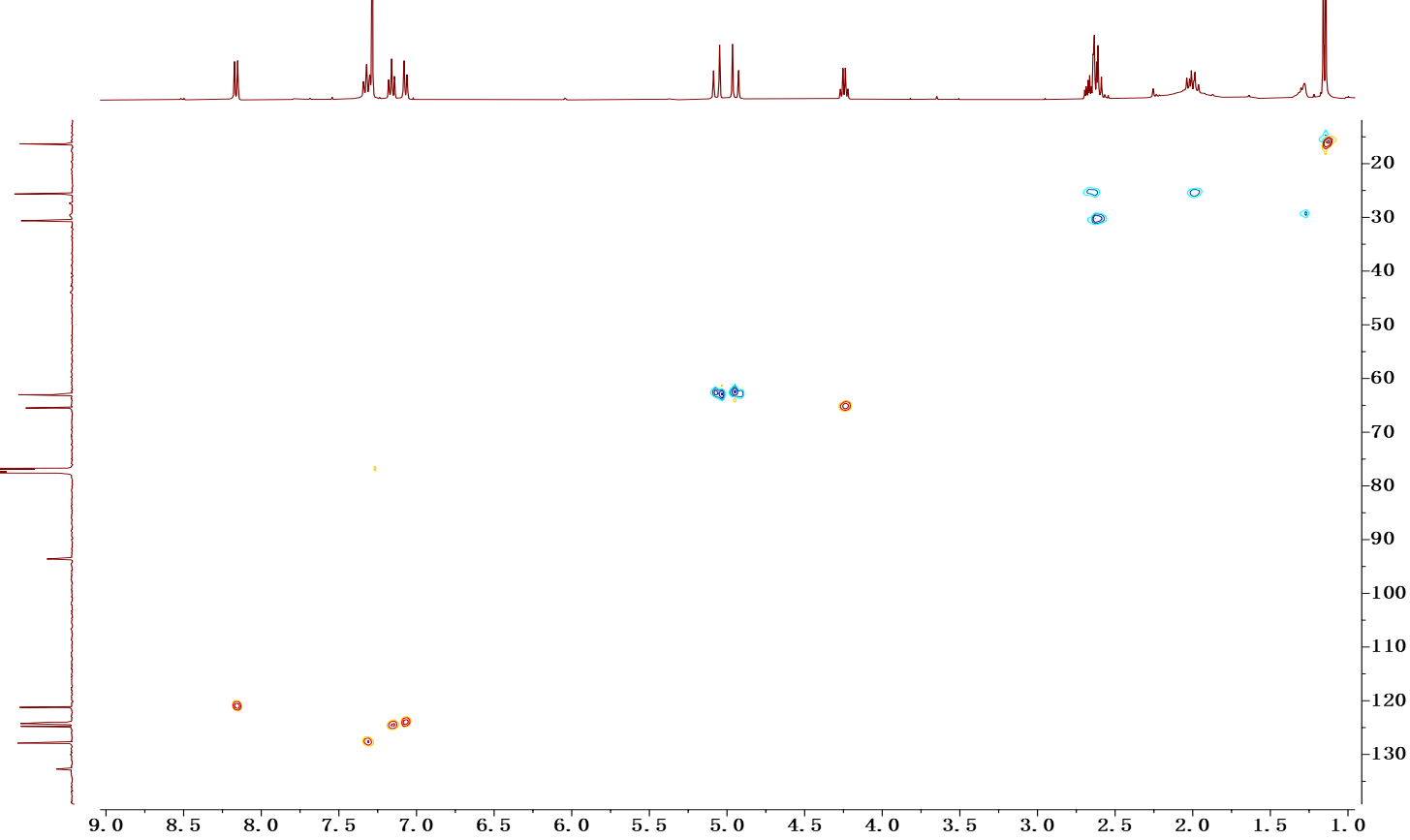


**Figure S4.** The HSQC spectrum of compound **1** in CDCl_3_.


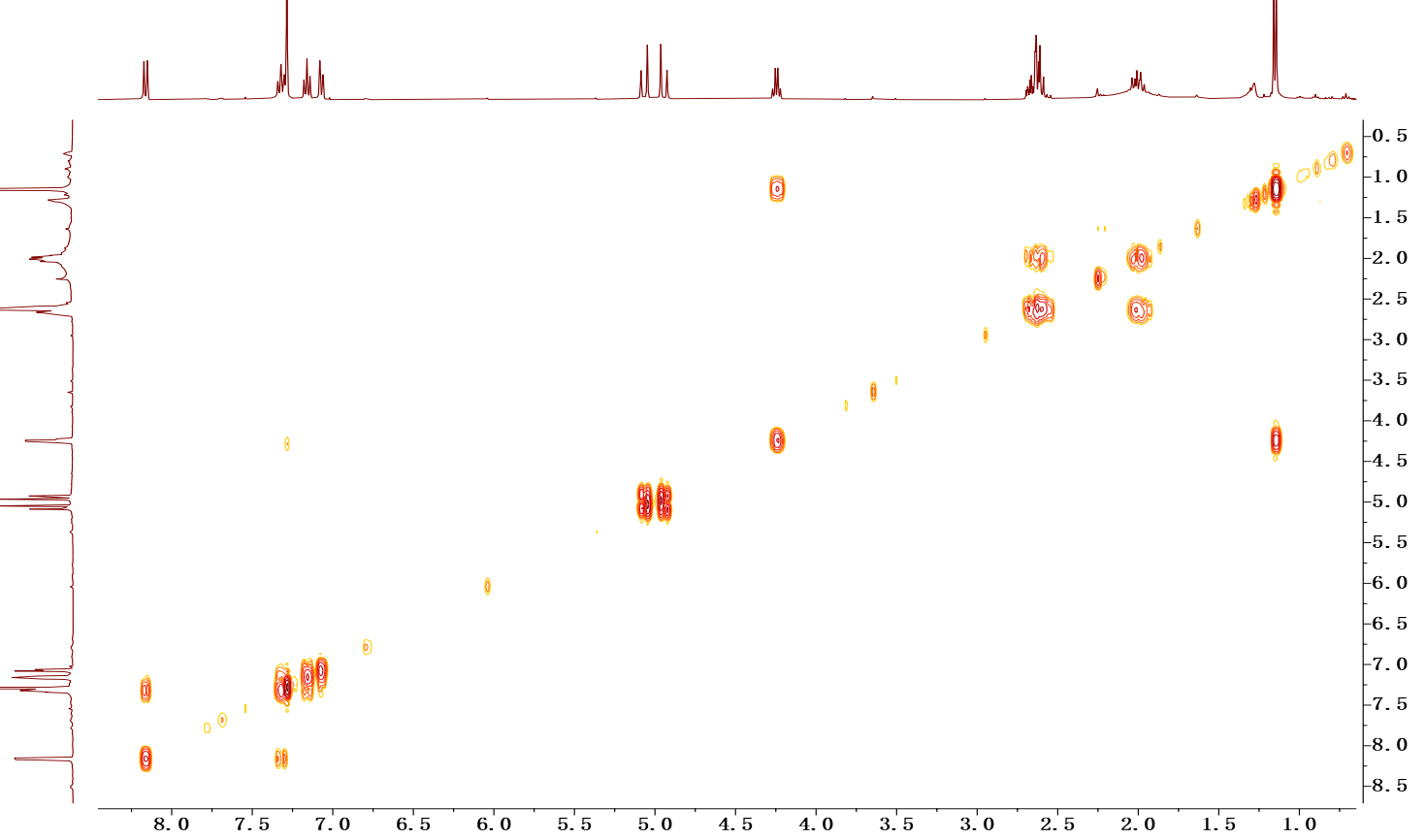


**Figure S5.** The ^1^H-^1^H COSY spectrum of compound **1** in CDCl_3_.


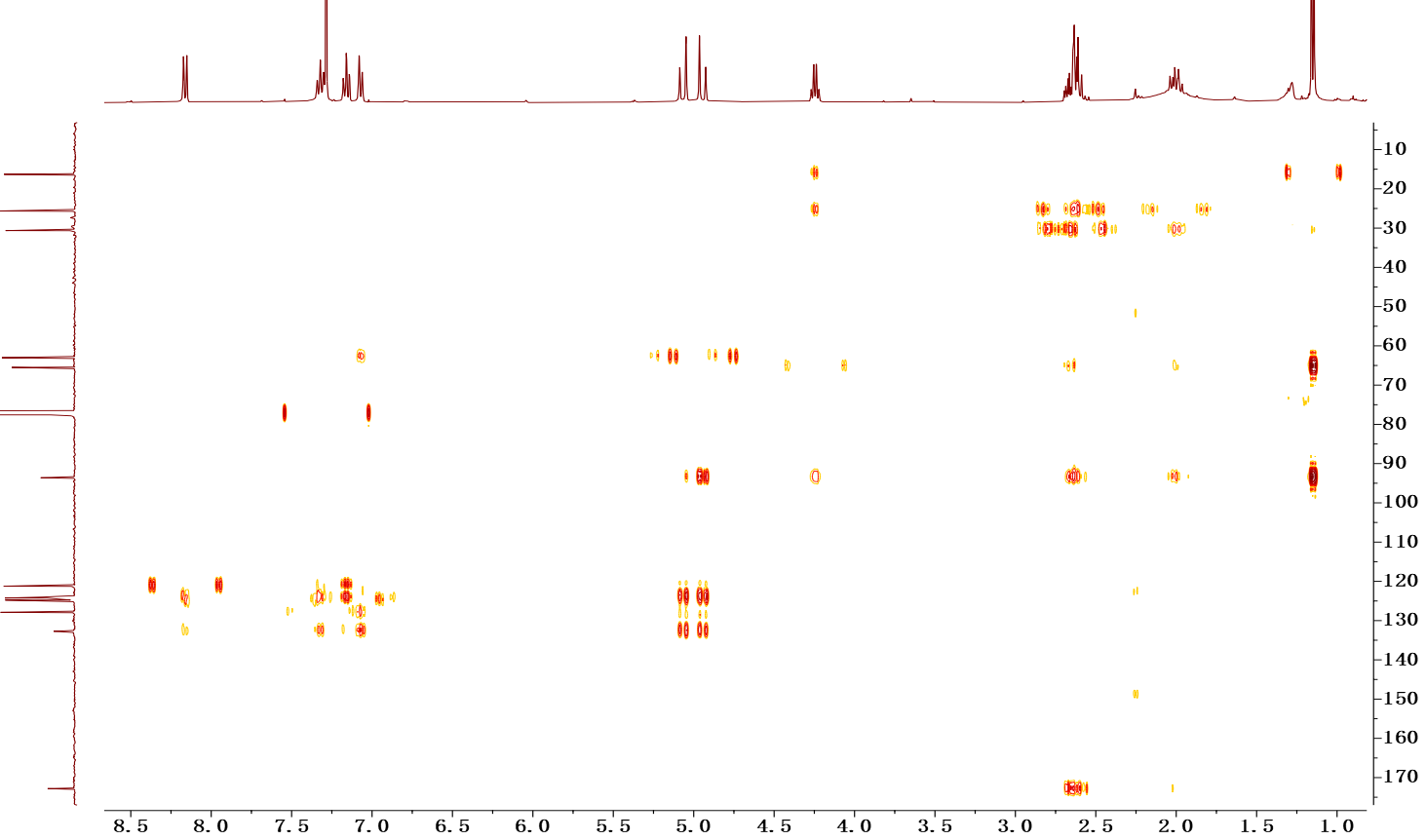


**Figure S6.** The HMBC spectrum of compound **1** in CDCl_3_.


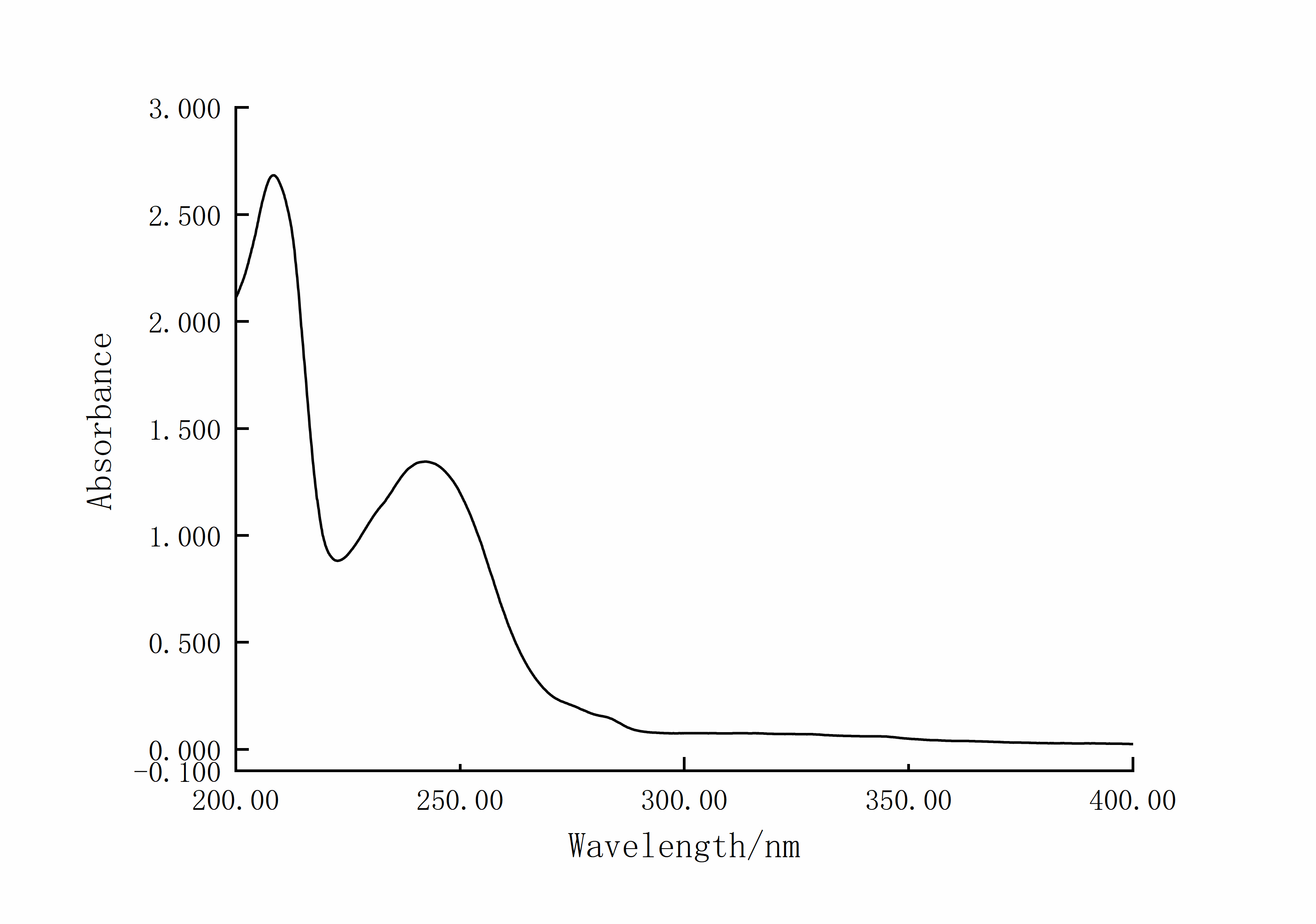


**Figure S7.** The UV spectrum of compound **1**.





**Figure S8.** The IR spectrum of compound **1**.


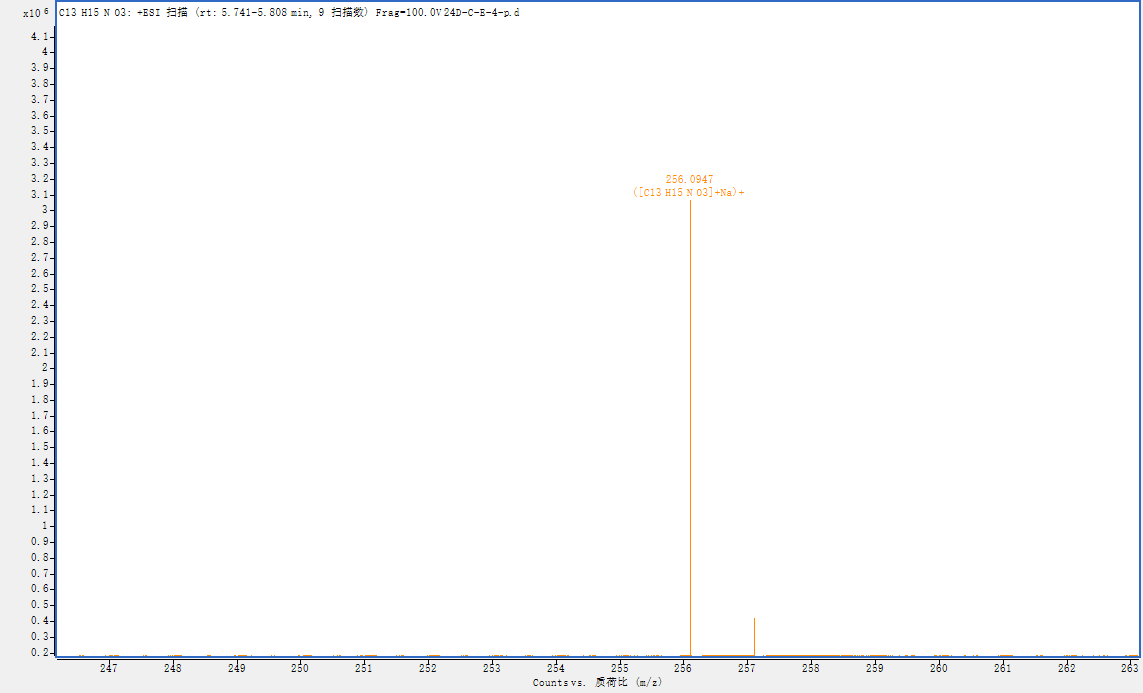


| SPECTRUM - simulation : |  |  |  |  |
| --- | --- | --- | --- | --- |
|  |  |  |  |  |
| *m/z* | Theo. Mass | Delta (ppm) | RDB equiv. | Composition |
| 256.0947 | 256.0950 | 1.17 | 7 | C_13_H_15_NO_3_Na |

**Figure S9.** The HR-ESIMS spectrum of compound **2**.


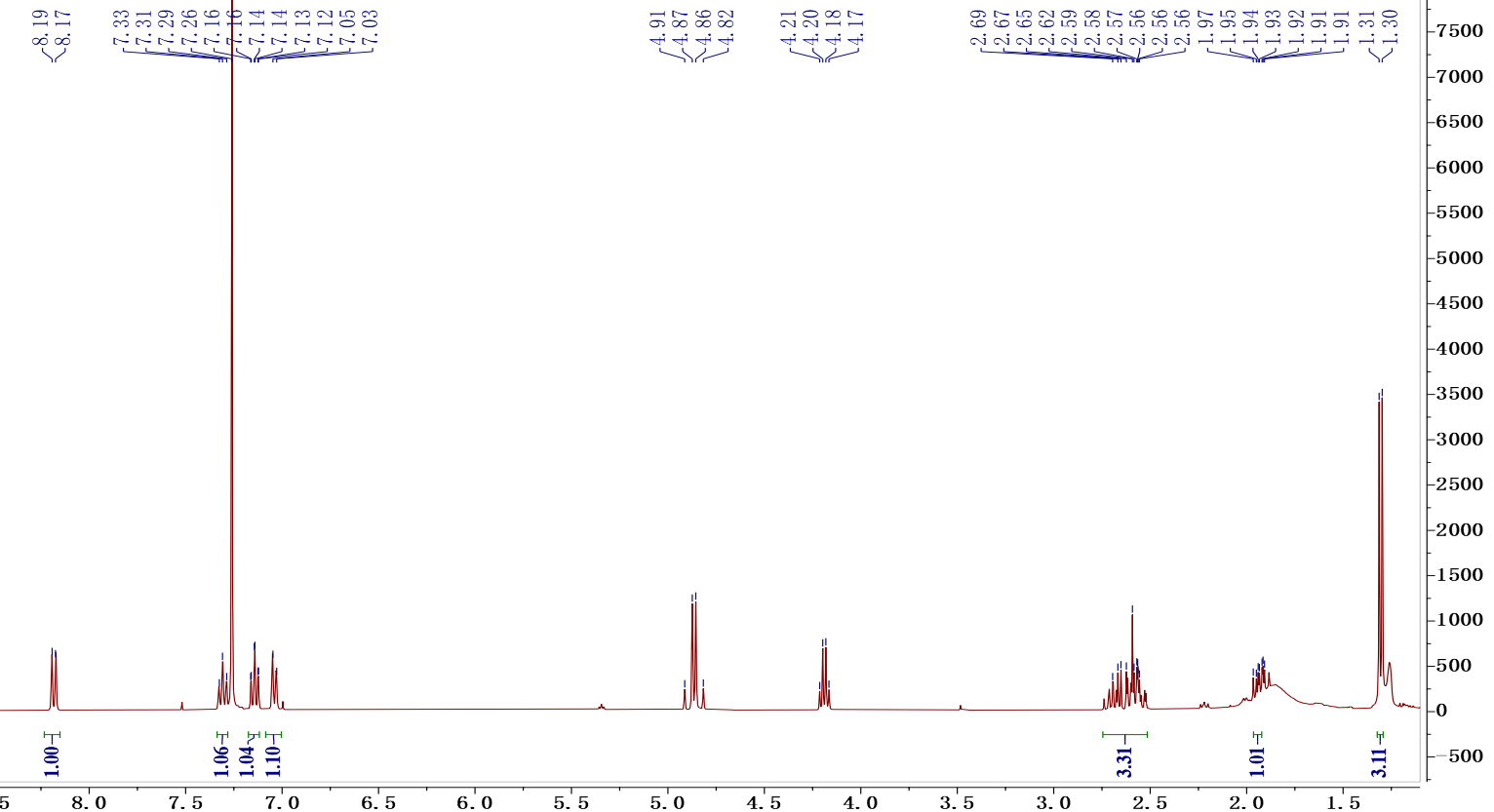


**Figure S10.** The ^1^H NMR (400 MHz) spectrum of compound **2** in CDCl_3_.


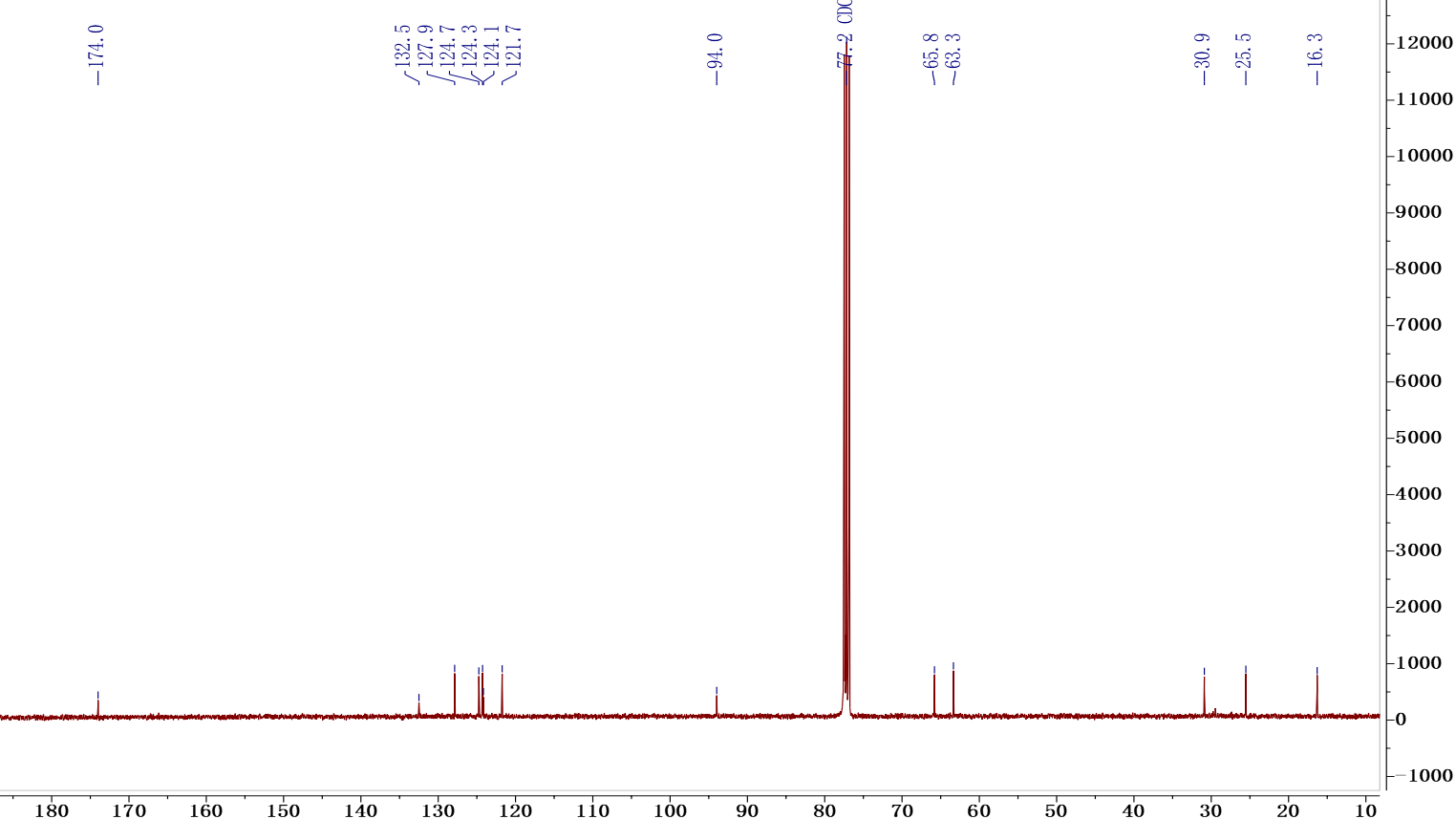


**Figure S11.** The ^13^C NMR (100 MHz) spectrum of compound **2** in CDCl_3_.


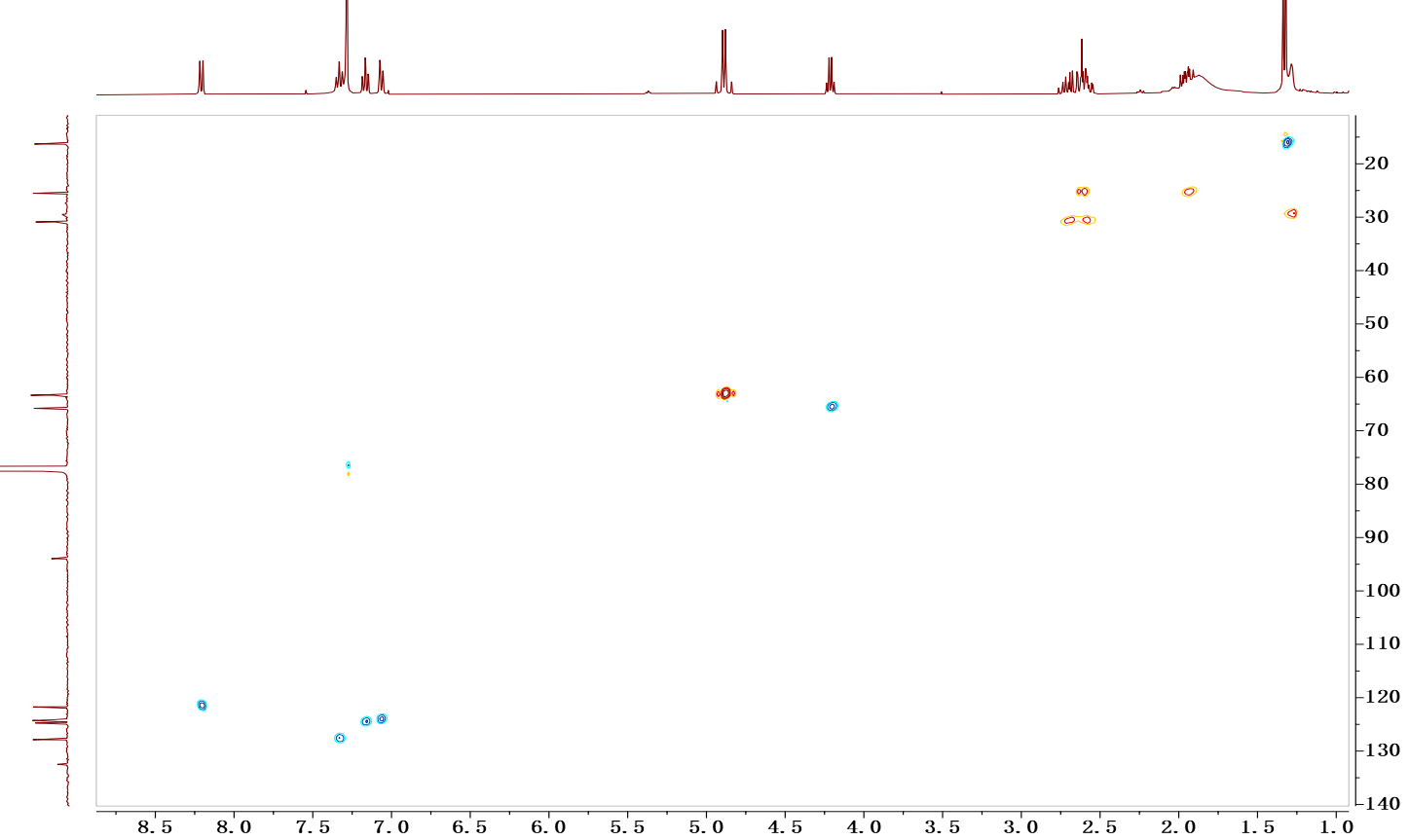


**Figure S12.** The HSQC spectrum of compound **2** in CDCl_3_.


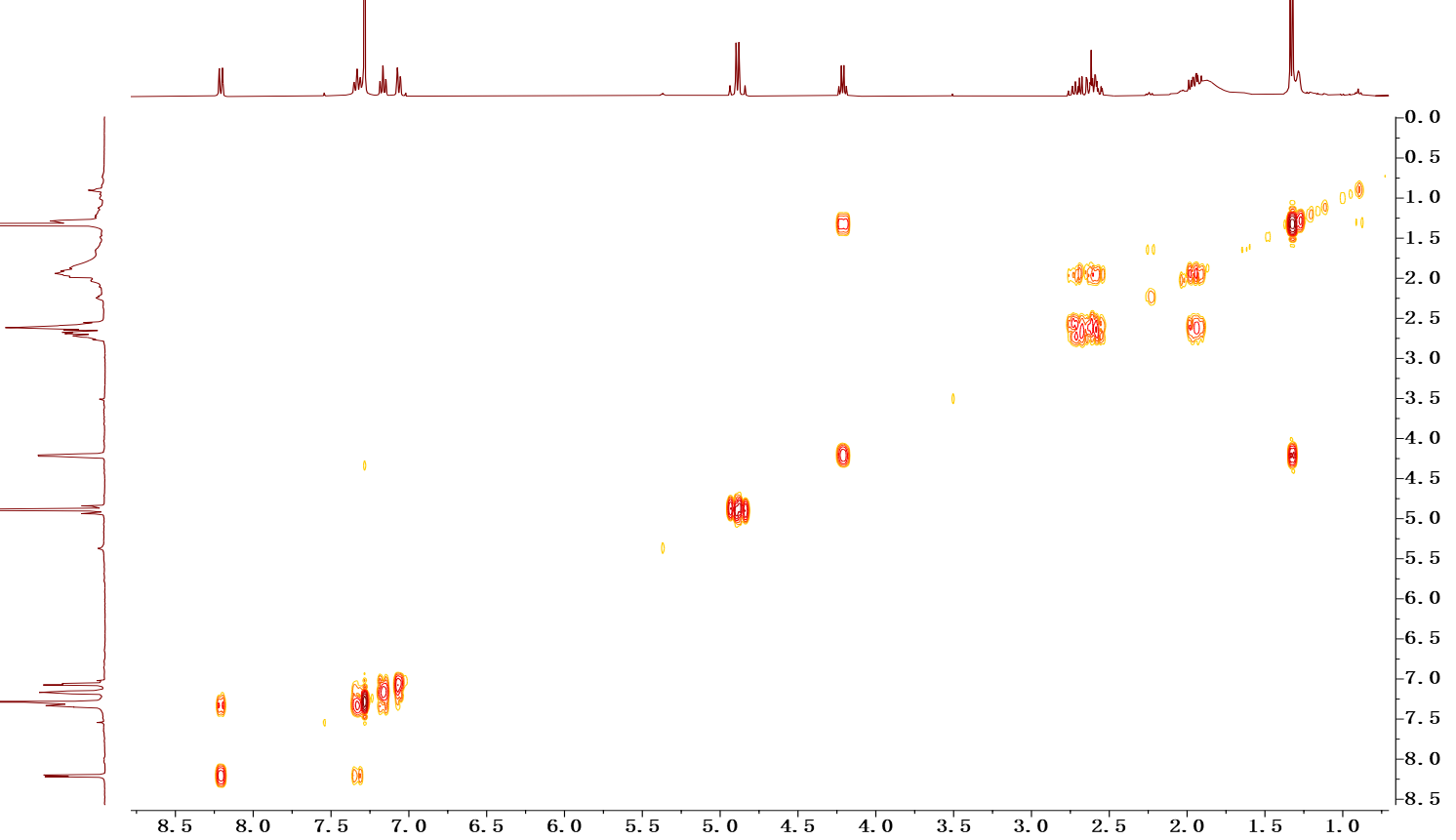


**Figure S13.** The ^1^H-^1^H COSY spectrum of compound **2** in CDCl_3_.

**
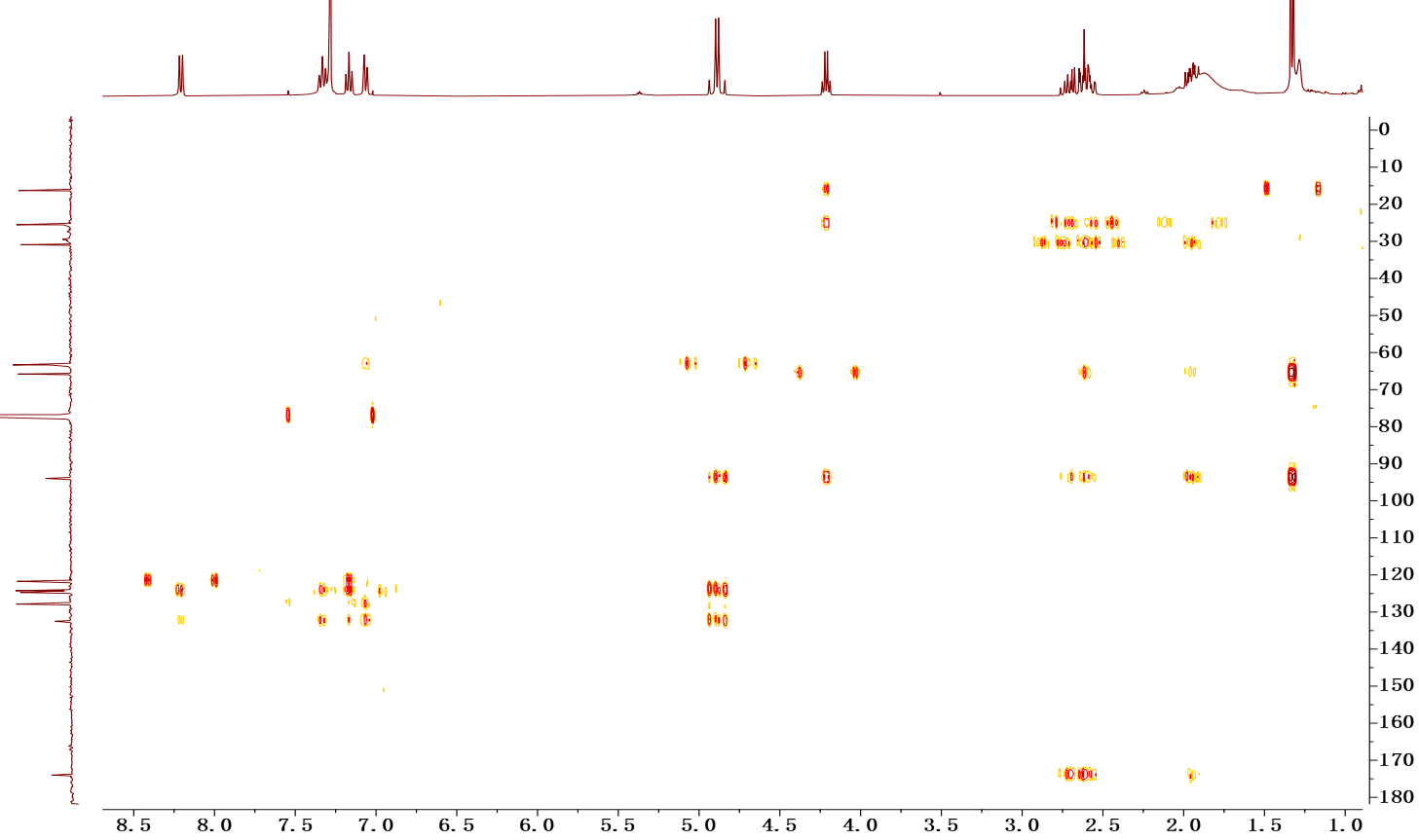
**

**Figure S14.** The HMBC spectrum of compound **2** in CDCl_3_.





**Figure S15.** The UV spectrum of compound **2**.


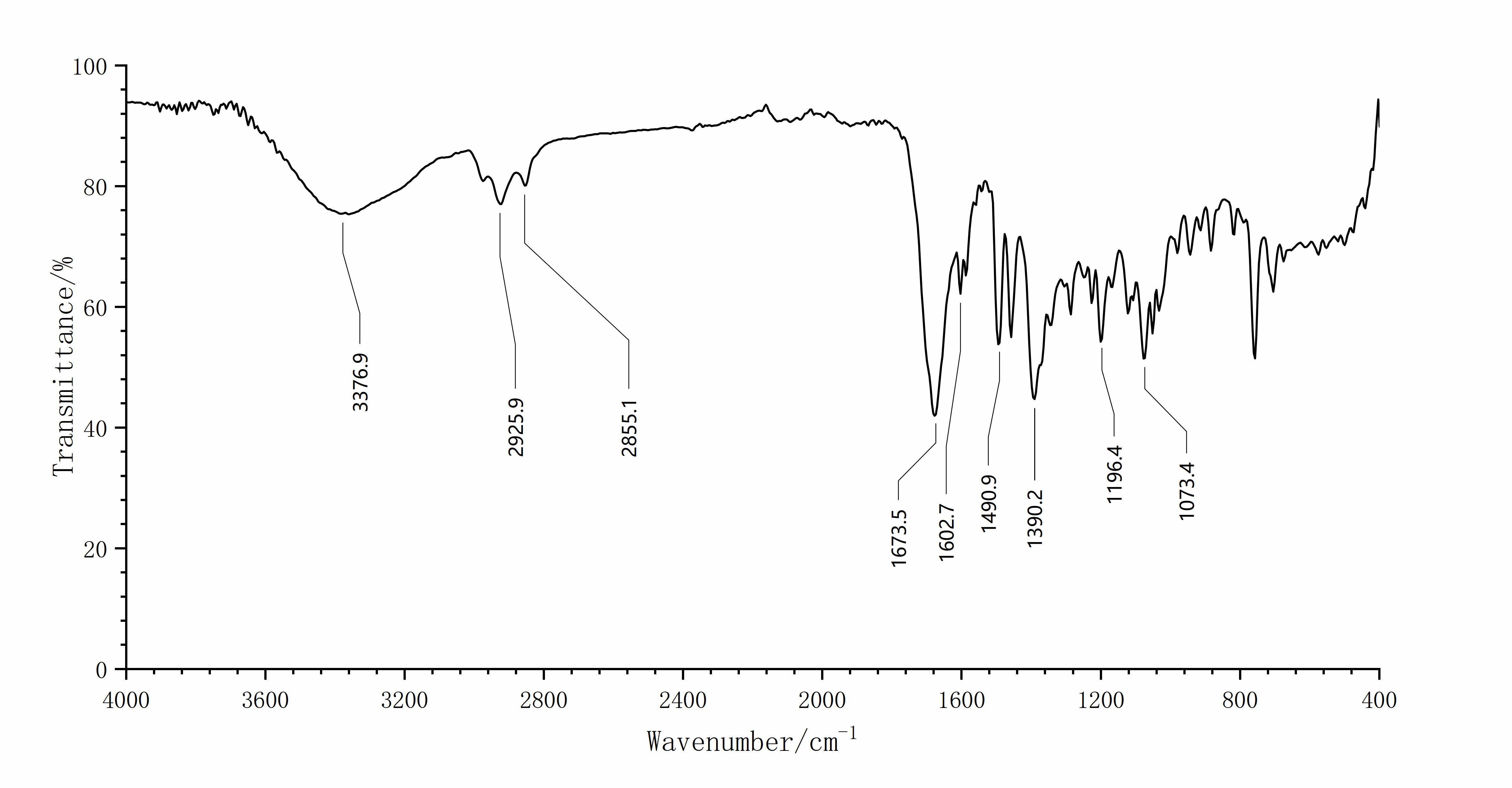


**Figure S16.** The IR spectrum of compound **2**.

**Figure S17.** ^1^H NMR (400 MHz) spectrum of (*S*)-MTPA esters **2** in pyridine-*d*_5_.

**Figure S18.** The ^1^H-^1^H COSY spectrum of (*S*)-MTPA esters **2** in pyridine-*d*_5_.

**Figure S19.** ^1^H NMR (400 MHz) spectrum of (*R*)-MTPA esters **2** in pyridine-*d*_5_.

**Figure S20.** The ^1^H-^1^H COSY spectrum of (*R*)-MTPA esters **2** in pyridine-*d*_5_.


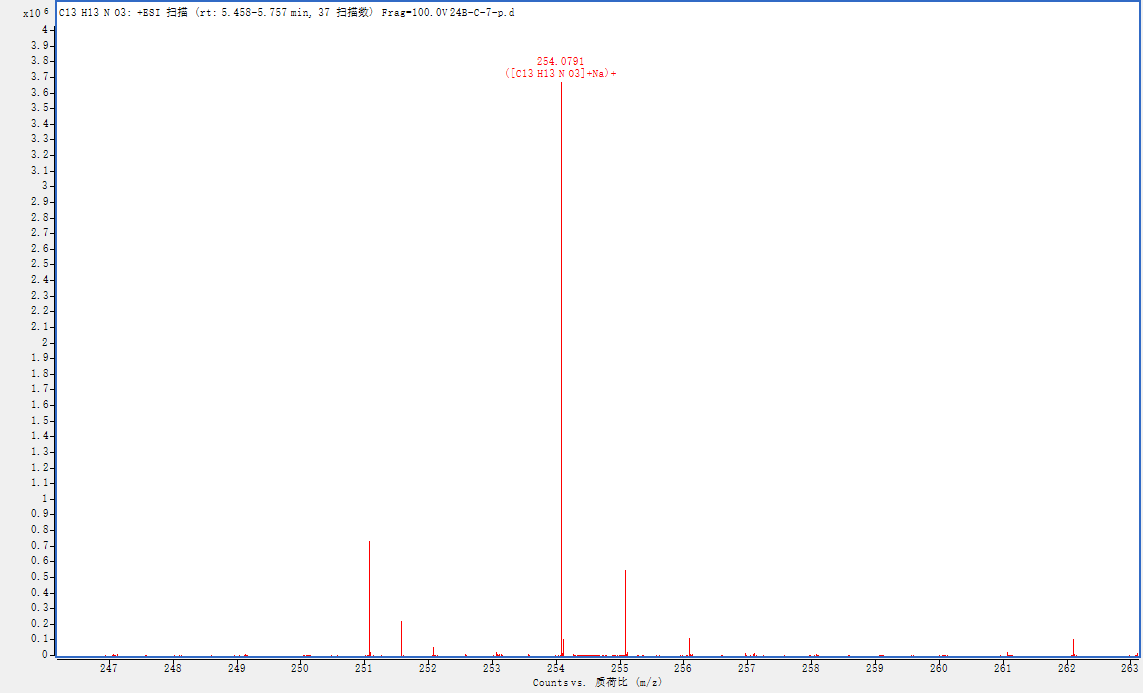


| SPECTRUM - simulation : |  |  |  |  |
| --- | --- | --- | --- | --- |
|  |  |  |  |  |
| m/z | Theo. Mass | Delta (ppm) | RDB equiv. | Composition |
| 254.0791 | 254.0730 | 1.15 | 8 | C_13_ H_13_NO_3_ Na |

**Figure S21.** The HR-ESIMS spectrum of compound **3**.


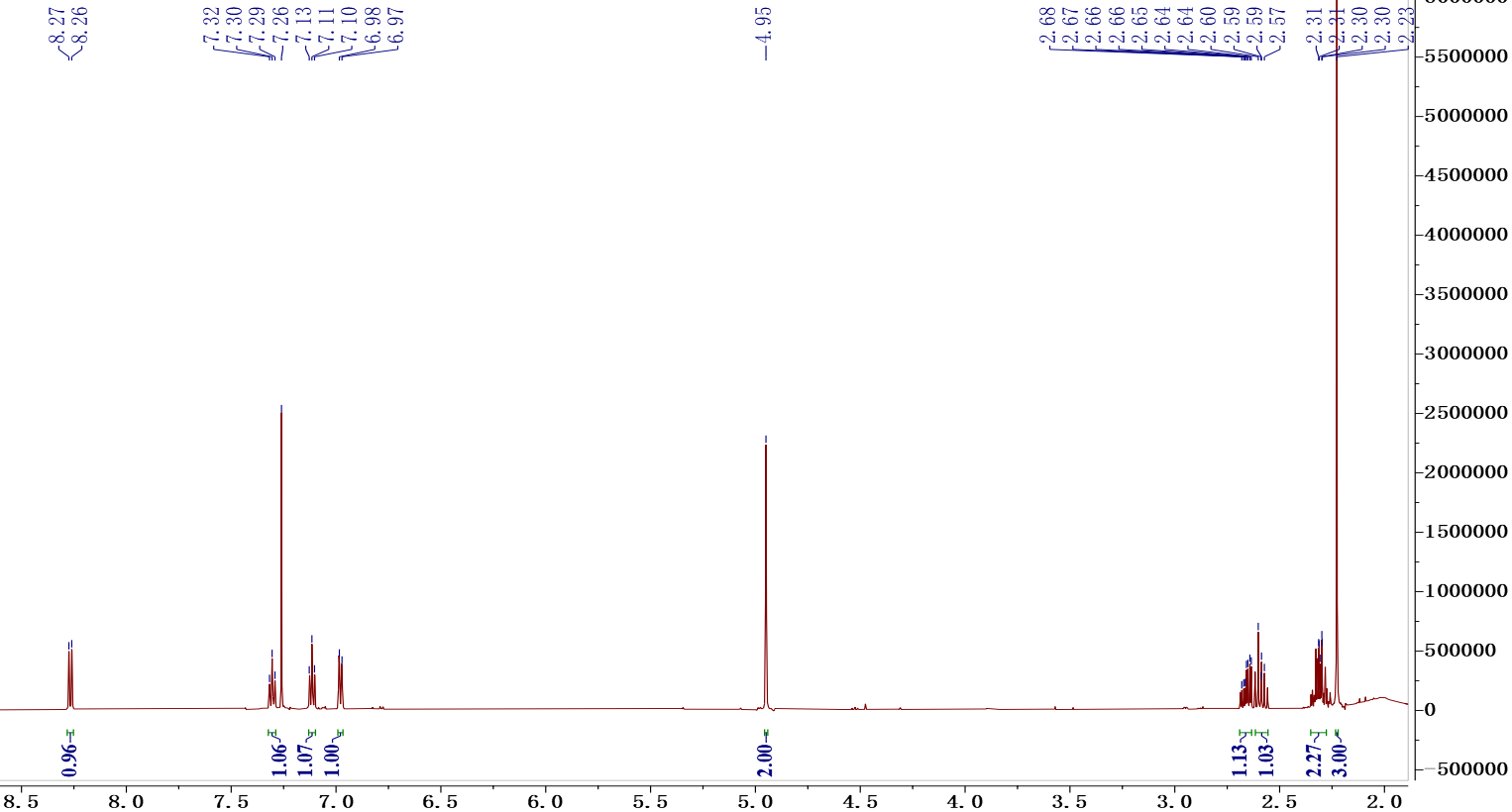


**Figure S22.** The ^1^H NMR (400 MHz) spectrum of compound **3** in CDCl_3_.


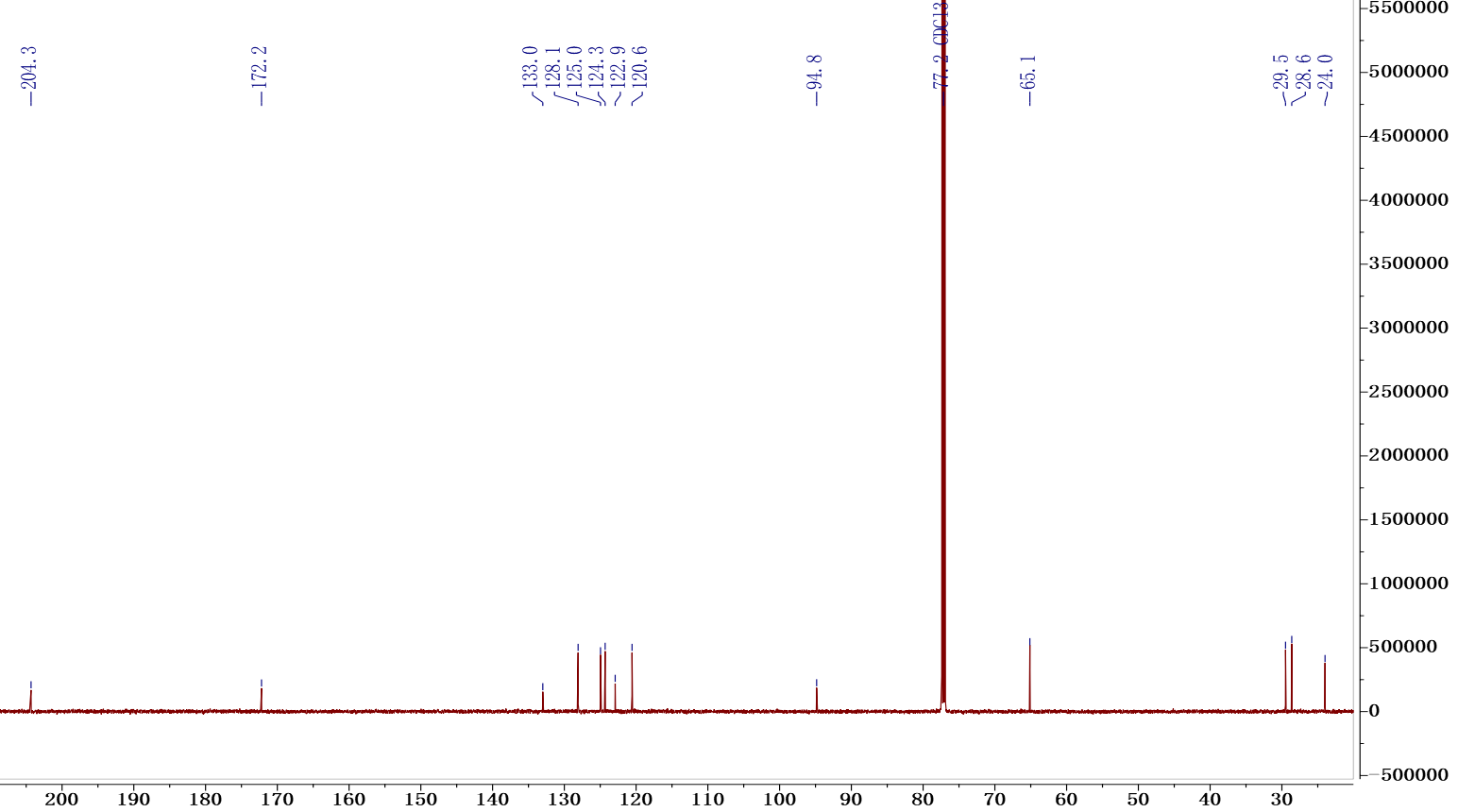


**Figure S23.** The ^13^C NMR (100 MHz) spectrum of compound **3** in CDCl_3_.


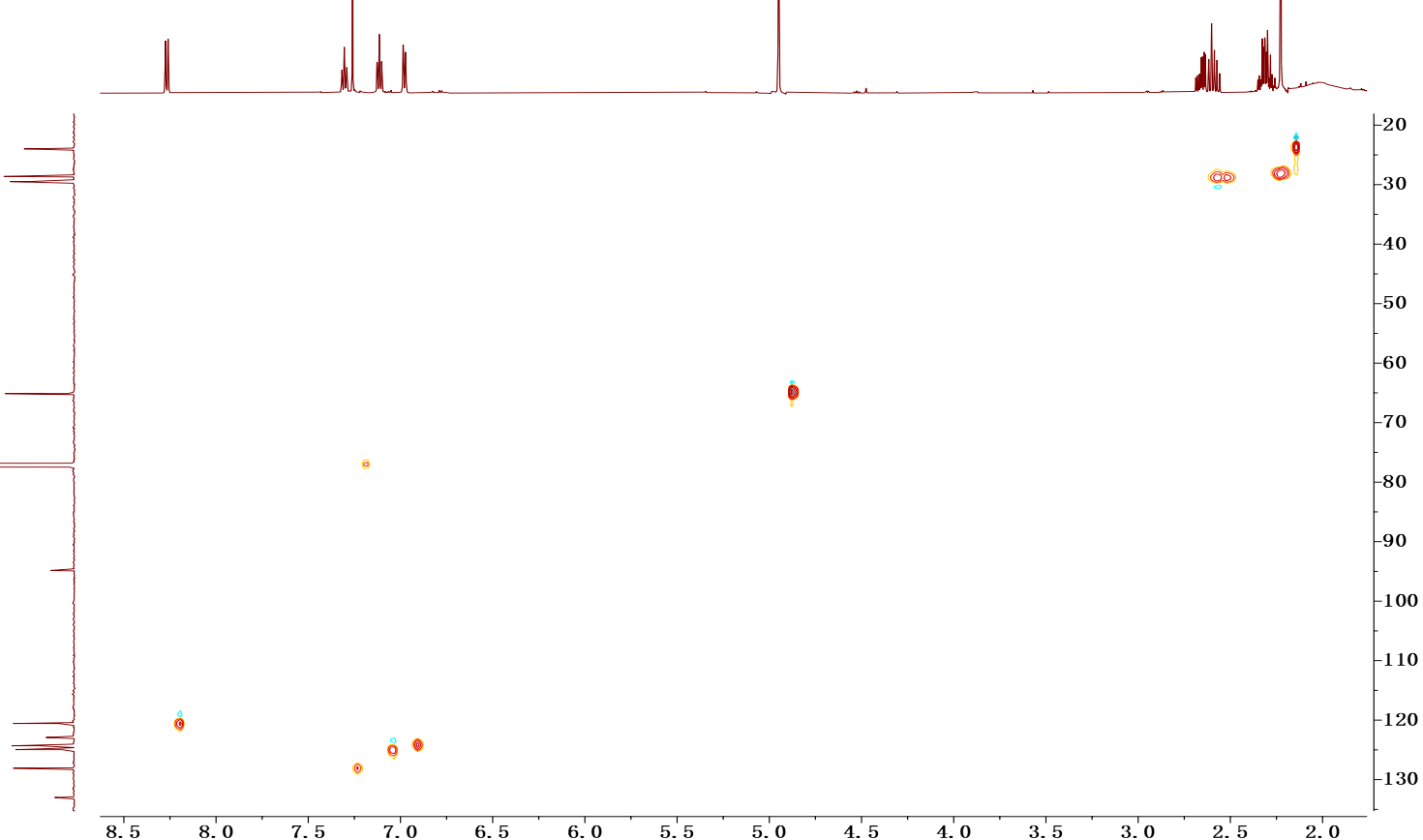


**Figure S24.** The HSQC spectrum of compound **3** in CDCl_3_.


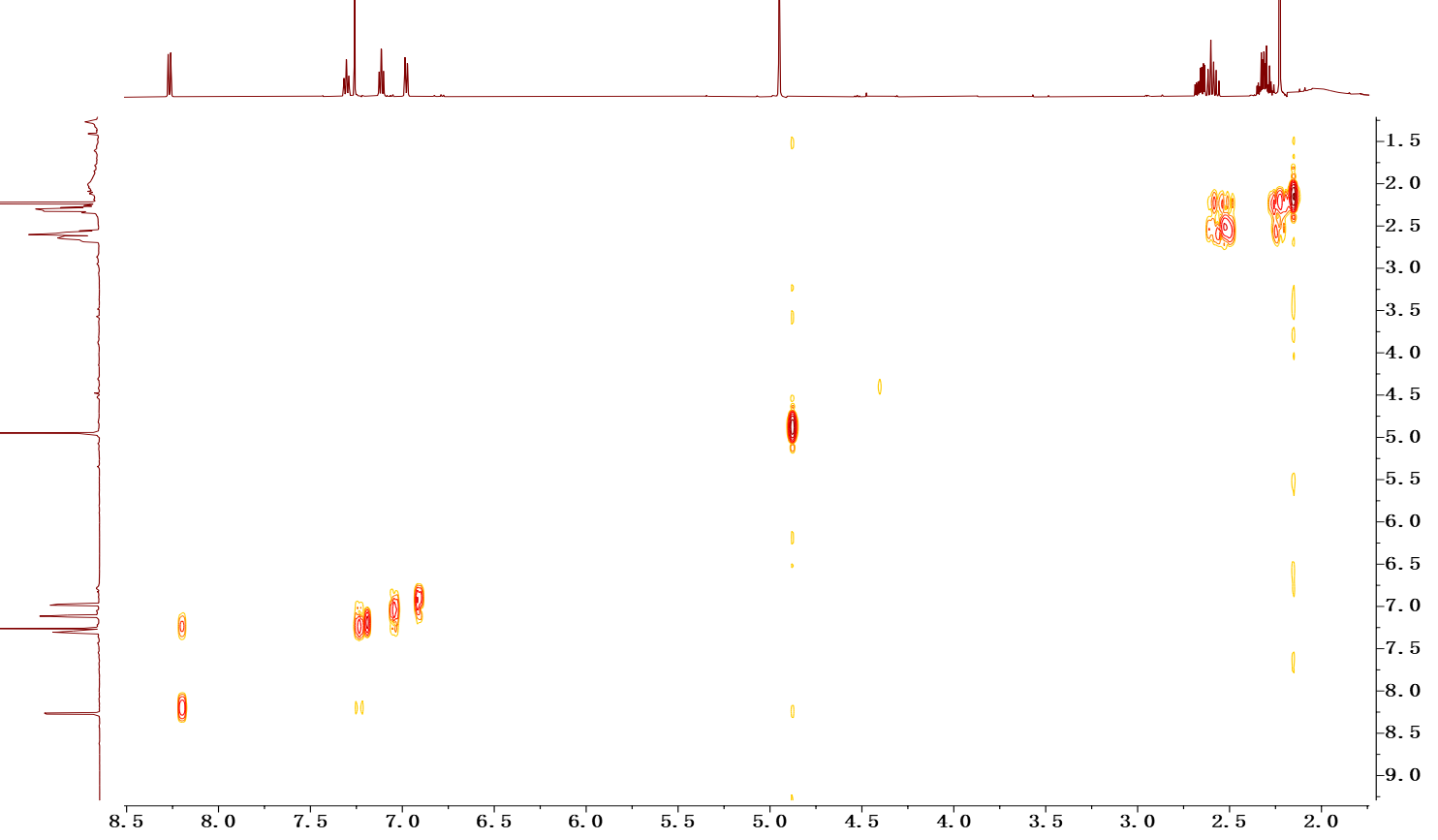
**Figure S25.** The ^1^H-^1^H COSY spectrum of compound **3** in CDCl_3_.

**
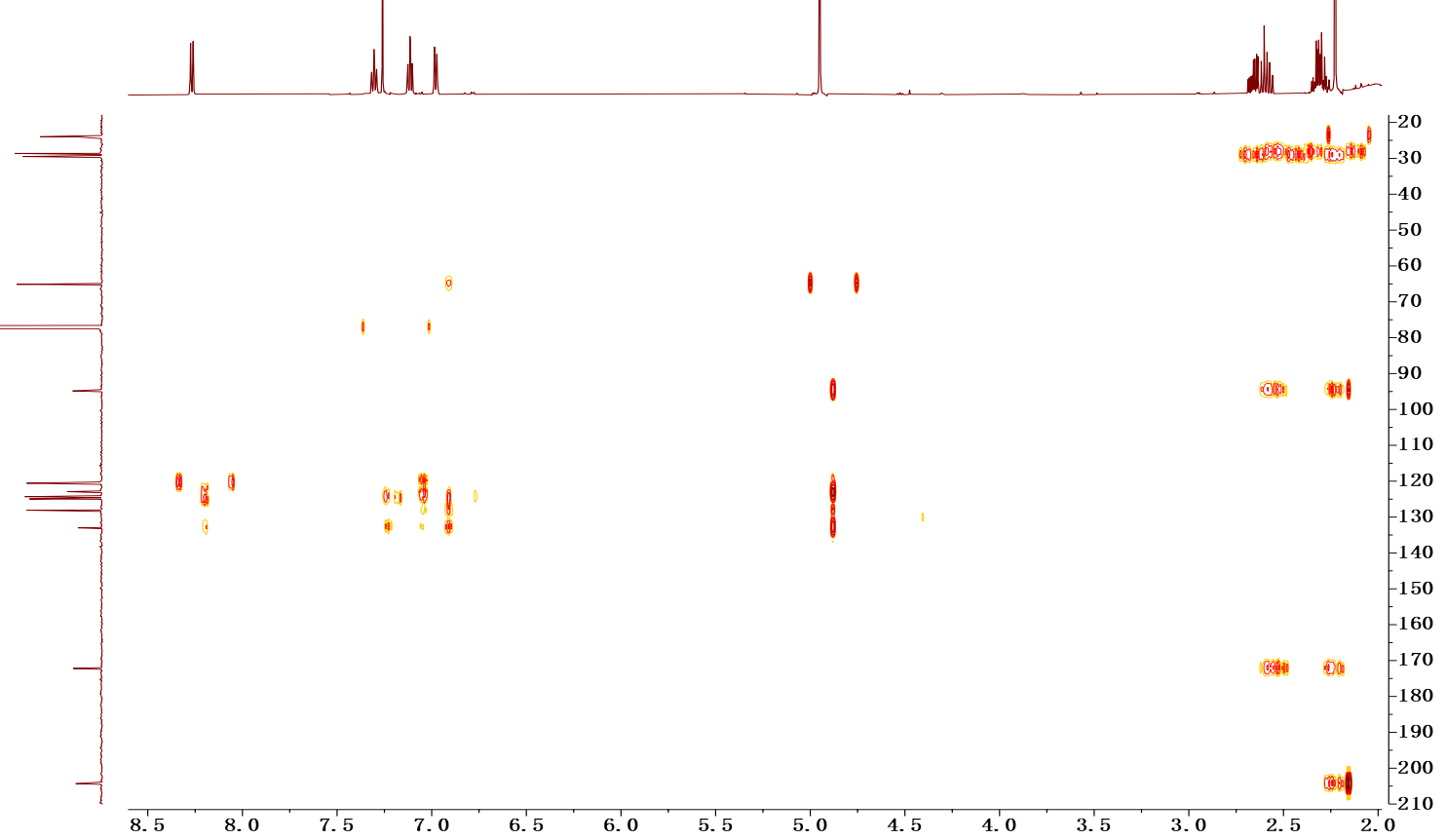
**

**Figure S26.** The HMBC spectrum of compound **3** in CDCl_3_.


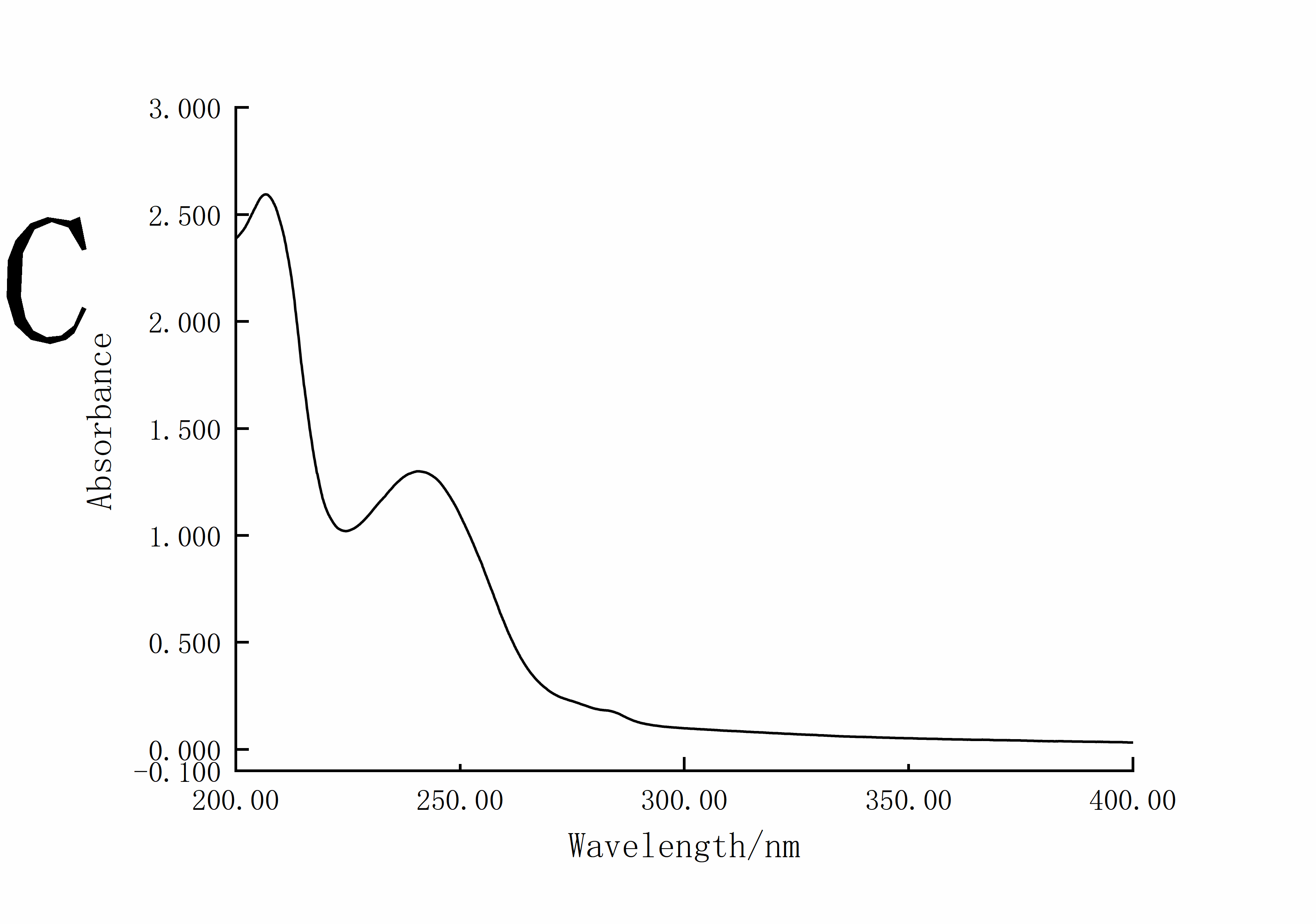


**Figure S27.** The UV spectrum of compound **3**.


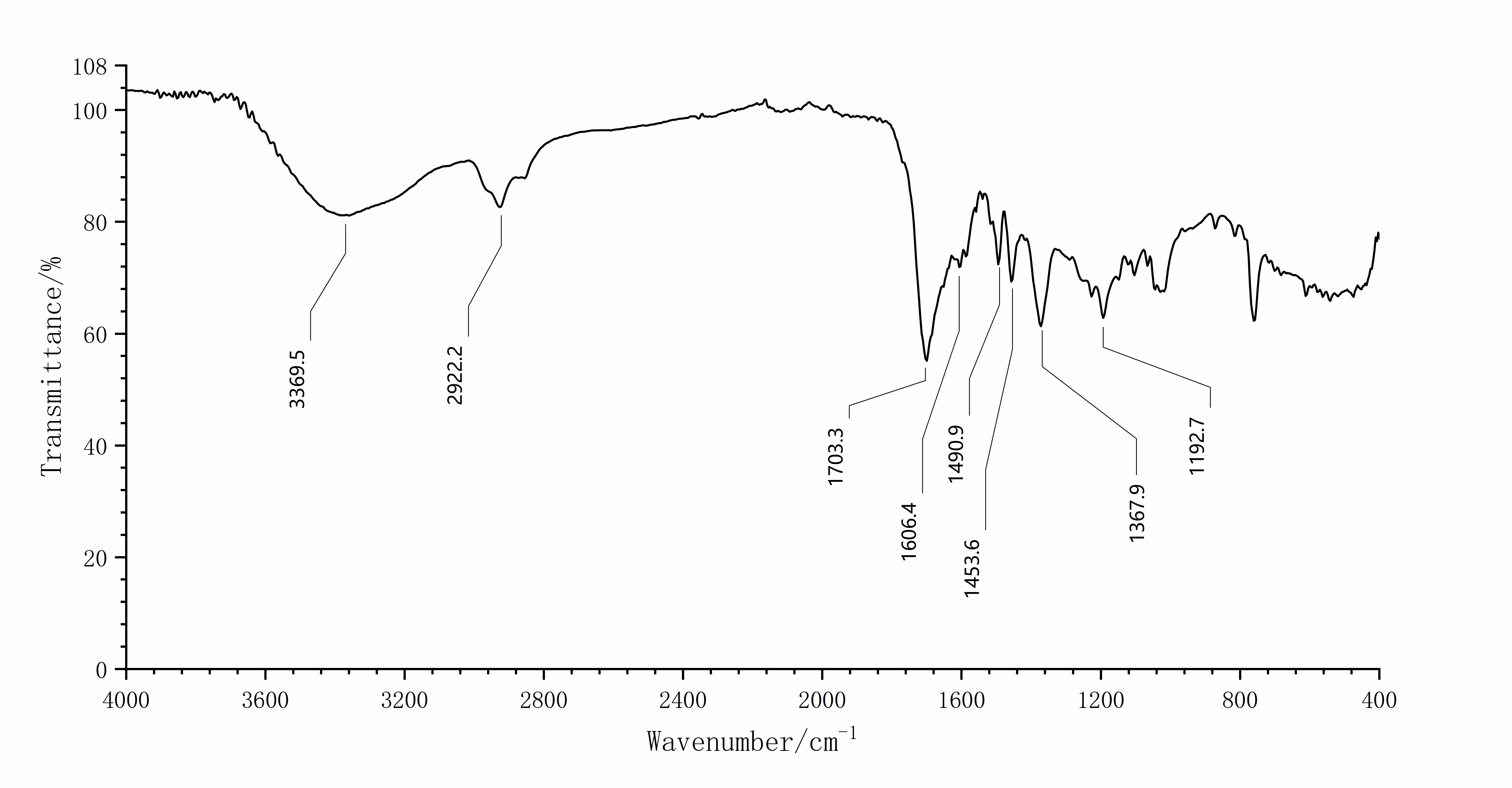


**Figure S28.** The IR spectrum of compound **3**.


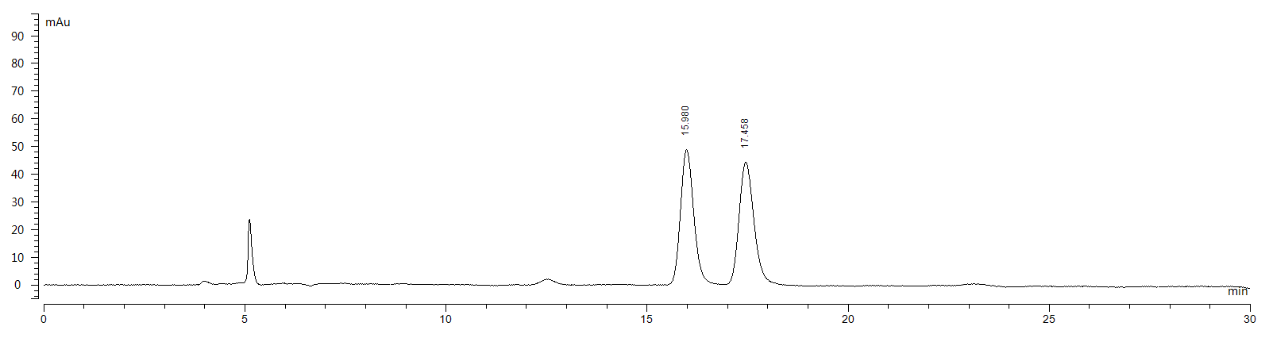


| No | Retention Time (min) | Peak Area (mAu*s) | Peak Width (min) | Peak Width at half height (min) | Peak Height (mAu) | Area percentage (%) |
| --- | --- | --- | --- | --- | --- | --- |
| 1 | 15.980 | 1080.33766 | 0.630 | 0.371 | 48.598 | 50.314 |
| 2 | 17.458 | 1066.83930 | 0.693 | 0.407 | 43.642 | 49.686 |

**Figure S29.** Chiral resolution liquid chromatogram of compound **3**.

**Table S1.** The energy and Boltzmann distribution of the optimized conformers of (5*R*, 1’*R*)-**1,** (5*S*, 1’*R*)-**2,** and (1’*R*)-**3**.

|  | Conformers | Relative energies (kcal/mol) | Boltzmann distribution (%) |
| --- | --- | --- | --- |
| (5*R*, 1’*R*)- **1** | **a** | 0 | 99.71% |
|  | **b** | 3.46259725 | 0.29% |
| (5*S*, 1’*R*)-**2** | **a** | 0 | 77.89% |
|  | **b** | 0.745544 | 22.11% |
| (1’*R*)-**3** | **a** | 0 | 62.95% |
|  | **b** | 0.313754734 | 37.05% |

**Table S2.** Cartesian coordinates of the low-energy reoptimized conformers of (5*R*, 1’*R*)-**1**.

| Conformer **a** | | | | Conformer **b** | | | |
| --- | --- | --- | --- | --- | --- | --- | --- |
| 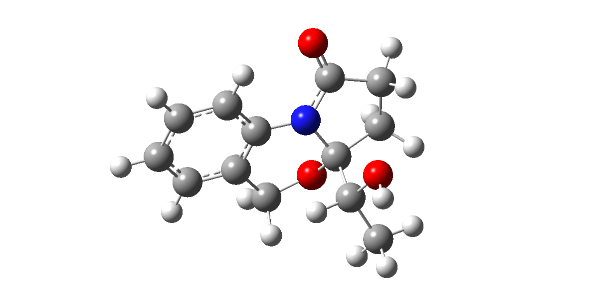 | | | | 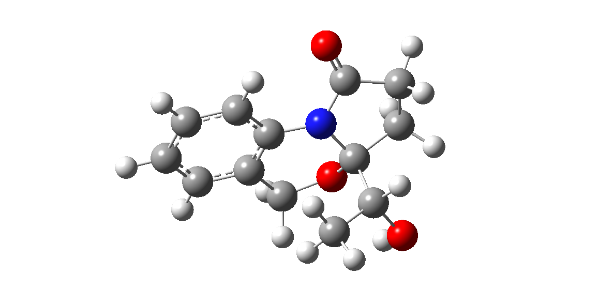 | | | |
| Atom | X axis  (Å) | Y axis  (Å) | Z axis  (Å) | Atom | X axis  (Å) | Y axis  (Å) | Z axis  (Å) |
| C | -3.451522 | 0.826038 | 0.652334 | C | -3.417919 | 0.790465 | 0.53787 |
| C | -3.833934 | -0.474322 | 0.303984 | C | -3.762855 | -0.461968 | 0.017056 |
| C | -2.881126 | -1.351566 | -0.216306 | C | -2.767824 | -1.273053 | -0.529283 |
| C | -1.550977 | -0.952149 | -0.395194 | C | -1.43208 | -0.85301 | -0.567425 |
| C | -1.169714 | 0.356526 | -0.027154 | C | -1.091101 | 0.404304 | -0.030816 |
| C | -2.130492 | 1.245009 | 0.492978 | C | -2.092957 | 1.225953 | 0.520176 |
| C | -0.539357 | -1.915732 | -0.974332 | C | -0.381846 | -1.753034 | -1.171684 |
| O | 0.693611 | -1.297268 | -1.319465 | O | 0.90285 | -1.14998 | -1.295135 |
| C | 1.181271 | -0.354761 | -0.392433 | C | 1.309311 | -0.223775 | -0.305842 |
| N | 0.183639 | 0.714554 | -0.202984 | N | 0.268991 | 0.788097 | -0.067215 |
| C | 2.372386 | 0.392505 | -1.021255 | C | 2.472345 | 0.604834 | -0.882689 |
| C | 2.201655 | 1.848291 | -0.584465 | C | 2.268532 | 1.999173 | -0.290541 |
| C | 0.721391 | 1.984829 | -0.265949 | C | 0.770419 | 2.080448 | -0.036494 |
| O | 0.11707 | 3.030037 | -0.094536 | O | 0.12668 | 3.093944 | 0.164862 |
| C | 1.515787 | -1.02402 | 0.970366 | C | 1.768516 | -0.950354 | 1.000092 |
| C | 2.440519 | -2.231242 | 0.837652 | C | 0.688794 | -1.500526 | 1.933699 |
| O | 2.08823 | -0.006075 | 1.776599 | O | 2.70078 | -1.953853 | 0.633161 |
| H | -4.188095 | 1.52452 | 1.05765 | H | -4.186873 | 1.436685 | 0.968659 |
| H | -4.868509 | -0.801043 | 0.432181 | H | -4.800444 | -0.803256 | 0.035123 |
| H | -3.172691 | -2.366751 | -0.501651 | H | -3.027982 | -2.251624 | -0.943518 |
| H | -1.827026 | 2.254953 | 0.75739 | H | -1.819996 | 2.199077 | 0.920398 |
| H | -0.376167 | -2.761823 | -0.278438 | H | -0.316763 | -2.697638 | -0.599763 |
| H | -0.924957 | -2.349352 | -1.909897 | H | -0.674287 | -2.02961 | -2.197085 |
| H | 3.336518 | -0.042332 | -0.732067 | H | 3.439917 | 0.143732 | -0.650872 |
| H | 2.264196 | 0.293858 | -2.111152 | H | 2.34707 | 0.62569 | -1.974798 |
| H | 2.483269 | 2.587207 | -1.348144 | H | 2.577285 | 2.823166 | -0.948376 |
| H | 2.766546 | 2.073395 | 0.332629 | H | 2.793434 | 2.136324 | 0.67011 |
| H | 0.561131 | -1.357532 | 1.418896 | H | 2.340346 | -0.201383 | 1.574512 |
| H | 3.411189 | -1.943845 | 0.405902 | H | -0.003117 | -0.71398 | 2.267456 |
| H | 2.627742 | -2.665926 | 1.832406 | H | 0.105822 | -2.309541 | 1.471912 |
| H | 1.996591 | -3.013443 | 0.205217 | H | 1.184209 | -1.917037 | 2.82397 |
| H | 2.178182 | -0.343761 | 2.677832 | H | 2.327955 | -2.414909 | -0.135044 |

**Table S3.** Cartesian coordinates of the low-energy reoptimized conformers of (5*S*, 1’*R*)-**2**.

| Conformer **a** | | | | Conformer **b** | | | |
| --- | --- | --- | --- | --- | --- | --- | --- |
| 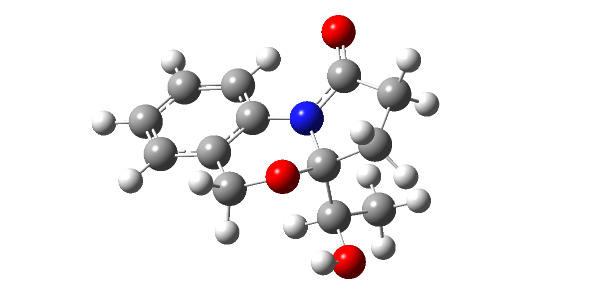 | | | | 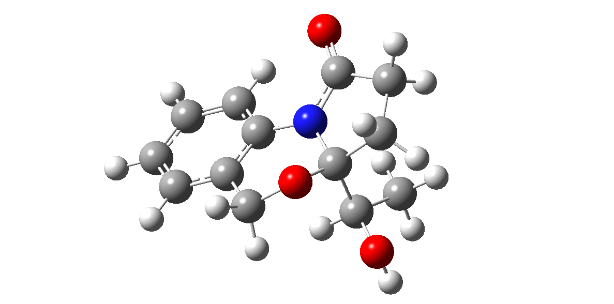 | | | |
| Atom | X axis  (Å) | Y axis  (Å) | Z axis  (Å) | Atom | X axis  (Å) | Y axis  (Å) | Z axis  (Å) |
| C | 3.387768 | 0.82562 | 0.713795 | C | 3.378282 | 0.824113 | 0.726208 |
| C | 3.788919 | -0.434466 | 0.288596 | C | 3.78349 | -0.430025 | 0.288018 |
| C | 2.861091 | -1.290646 | -0.28073 | C | 2.859363 | -1.281524 | -0.295254 |
| C | 1.537473 | -0.901253 | -0.443445 | C | 1.535406 | -0.894386 | -0.457895 |
| C | 1.144001 | 0.365556 | -0.01016 | C | 1.137517 | 0.367575 | -0.010266 |
| C | 2.070409 | 1.228258 | 0.573368 | C | 2.060163 | 1.22539 | 0.58585 |
| C | 0.541957 | -1.860264 | -1.042914 | C | 0.538598 | -1.84192 | -1.077163 |
| O | -0.715583 | -1.263452 | -1.33543 | O | -0.694853 | -1.217902 | -1.389363 |
| C | -1.186451 | -0.358657 | -0.353471 | C | -1.18434 | -0.368127 | -0.378292 |
| N | -0.21357 | 0.711298 | -0.170191 | N | -0.221703 | 0.711434 | -0.164756 |
| C | -2.421728 | 0.349978 | -0.915507 | C | -2.430391 | 0.344186 | -0.914926 |
| C | -2.220144 | 1.824956 | -0.581798 | C | -2.232919 | 1.817493 | -0.569096 |
| C | -0.735039 | 1.967754 | -0.33589 | C | -0.749179 | 1.965001 | -0.315794 |
| O | -0.114028 | 3.010344 | -0.276841 | O | -0.135353 | 3.011852 | -0.241449 |
| C | -1.445451 | -1.114423 | 0.970908 | C | -1.42205 | -1.131892 | 0.943757 |
| C | -2.005575 | -0.240074 | 2.072585 | C | -1.965264 | -0.249612 | 2.056841 |
| O | -2.363033 | -2.168444 | 0.734656 | O | -2.306682 | -2.194082 | 0.628277 |
| H | 4.103636 | 1.500832 | 1.165483 | H | 4.091157 | 1.495665 | 1.188052 |
| H | 4.818577 | -0.748324 | 0.403199 | H | 4.813642 | -0.742665 | 0.402109 |
| H | 3.16552 | -2.276538 | -0.614287 | H | 3.167899 | -2.261886 | -0.641251 |
| H | 1.754323 | 2.204074 | 0.907825 | H | 1.74033 | 2.196599 | 0.930412 |
| H | 0.911265 | -2.239973 | -1.995031 | H | 0.922642 | -2.224203 | -2.022597 |
| H | 0.413742 | -2.722291 | -0.377081 | H | 0.375312 | -2.702152 | -0.417305 |
| H | -2.423897 | 0.196392 | -1.993399 | H | -2.445518 | 0.200713 | -1.993939 |
| H | -3.337985 | -0.070823 | -0.508873 | H | -3.340004 | -0.083969 | -0.501728 |
| H | -2.528873 | 2.503739 | -1.373996 | H | -2.539847 | 2.50119 | -1.357941 |
| H | -2.740771 | 2.122164 | 0.330905 | H | -2.758675 | 2.108162 | 0.342707 |
| H | -0.489937 | -1.526659 | 1.31713 | H | -0.461261 | -1.539432 | 1.277897 |
| H | -2.971729 | 0.181741 | 1.792208 | H | -2.937886 | 0.169244 | 1.79509 |
| H | -2.145426 | -0.838573 | 2.972554 | H | -2.0864 | -0.842149 | 2.965296 |
| H | -1.316472 | 0.570653 | 2.309747 | H | -1.2738 | 0.562285 | 2.284727 |
| H | -2.079206 | -2.63712 | -0.056148 | H | -2.472625 | -2.694003 | 1.430132 |

**Table S4.** Cartesian coordinates of the low-energy reoptimized conformers of (1’*R*)-**3**.

| Conformer **a** | | | | Conformer **b** | | | |
| --- | --- | --- | --- | --- | --- | --- | --- |
| 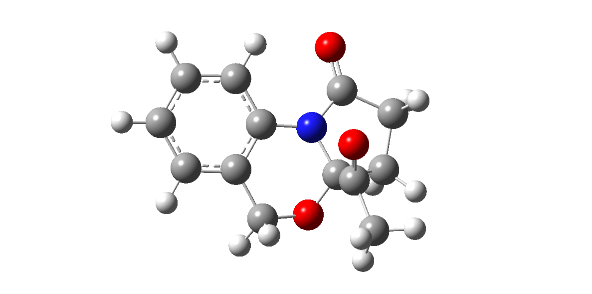 | | | | 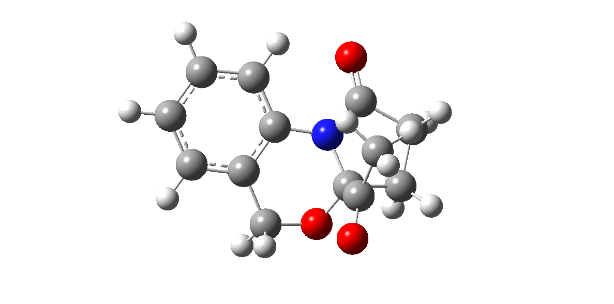 | | | |
| Atom | X axis  (Å) | Y axis  (Å) | Z axis  (Å) | Atom | X axis  (Å) | Y axis  (Å) | Z axis  (Å) |
| C | -3.413447 | 0.82694 | 0.508905 | C | -3.251382 | 1.08468 | 0.614377 |
| C | -3.766381 | -0.48666 | 0.180804 | C | -3.740074 | -0.159665 | 0.198203 |
| C | -2.784159 | -1.364352 | -0.281074 | C | -2.862862 | -1.09561 | -0.350607 |
| C | -1.452998 | -0.952408 | -0.416385 | C | -1.498477 | -0.810068 | -0.494464 |
| C | -1.102737 | 0.370143 | -0.067508 | C | -1.014053 | 0.439934 | -0.055162 |
| C | -2.092927 | 1.260493 | 0.387864 | C | -1.895662 | 1.388496 | 0.494578 |
| C | -0.404006 | -1.916842 | -0.923856 | C | -0.56907 | -1.82605 | -1.123321 |
| O | 0.799351 | -1.263222 | -1.321228 | O | 0.733562 | -1.309134 | -1.378667 |
| C | 1.248146 | -0.312627 | -0.394827 | C | 1.24433 | -0.48699 | -0.36743 |
| N | 0.25243 | 0.740729 | -0.20392 | N | 0.376475 | 0.67502 | -0.170895 |
| C | 2.46604 | 0.446006 | -0.955076 | C | 2.584927 | 0.124669 | -0.844965 |
| C | 2.306923 | 1.855455 | -0.383234 | C | 2.503871 | 1.612672 | -0.495441 |
| C | 0.809505 | 2.004134 | -0.145984 | C | 1.024231 | 1.893421 | -0.275437 |
| O | 0.215108 | 3.045646 | 0.064909 | O | 0.502614 | 2.988615 | -0.184116 |
| C | 1.616152 | -0.970926 | 0.976402 | C | 1.451378 | -1.290686 | 0.958905 |
| C | 2.449564 | -2.231576 | 0.93056 | C | 1.55872 | -0.513836 | 2.248236 |
| O | 1.293083 | -0.437366 | 2.013816 | O | 1.559609 | -2.495329 | 0.908277 |
| H | -4.173614 | 1.526135 | 0.866386 | H | -3.930625 | 1.825819 | 1.042799 |
| H | -4.801162 | -0.823228 | 0.277266 | H | -4.801788 | -0.397121 | 0.297293 |
| H | -3.052173 | -2.389604 | -0.552539 | H | -3.240602 | -2.06541 | -0.687586 |
| H | -1.811805 | 2.280519 | 0.637733 | H | -1.50745 | 2.353134 | 0.813655 |
| H | -0.188094 | -2.688972 | -0.158714 | H | -0.504792 | -2.731408 | -0.493956 |
| H | -0.767004 | -2.446282 | -1.816696 | H | -0.961275 | -2.135619 | -2.104113 |
| H | 3.419165 | -0.037813 | -0.704638 | H | 3.454286 | -0.388899 | -0.412842 |
| H | 2.357944 | 0.443045 | -2.050188 | H | 2.610619 | -0.02783 | -1.933097 |
| H | 2.663177 | 2.655197 | -1.046796 | H | 2.888882 | 2.276039 | -1.282467 |
| H | 2.821438 | 1.976928 | 0.584691 | H | 3.045316 | 1.868766 | 0.430294 |
| H | 3.493761 | -1.966137 | 1.16772 | H | 2.288344 | 0.306907 | 2.167696 |
| H | 2.104581 | -2.920983 | 1.714483 | H | 0.586998 | -0.05511 | 2.492419 |
| H | 2.428394 | -2.721056 | -0.050787 | H | 1.855218 | -1.193428 | 3.05739 |
